# Relevance of NADH Dehydrogenase and Alternative Two-Enzyme Systems for Growth of *Corynebacterium glutamicum* With Glucose, Lactate, and Acetate

**DOI:** 10.3389/fbioe.2020.621213

**Published:** 2021-01-20

**Authors:** Tomoya Maeda, Abigail Koch-Koerfges, Michael Bott

**Affiliations:** IBG-1: Biotechnology, Institute of Bio- and Geosciences, Forschungszentrum Jülich, Jülich, Germany

**Keywords:** NADH dehydrogenase, malate dehydrogenase, malate:quinone oxidoreductase, lactate dehydrogenase, NAD^+^/NADH ratio, respiratory chain, SugR

## Abstract

The oxidation of NADH with the concomitant reduction of a quinone is a crucial step in the metabolism of respiring cells. In this study, we analyzed the relevance of three different NADH oxidation systems in the actinobacterial model organism *Corynebacterium glutamicum* by characterizing defined mutants lacking the non-proton-pumping NADH dehydrogenase Ndh (Δ*ndh*) and/or one of the alternative NADH-oxidizing enzymes, L-lactate dehydrogenase LdhA (Δ*ldhA*) and malate dehydrogenase Mdh (Δ*mdh*). Together with the menaquinone-dependent L-lactate dehydrogenase LldD and malate:quinone oxidoreductase Mqo, the LdhA-LldD and Mdh-Mqo couples can functionally replace Ndh activity. In glucose minimal medium the Δ*ndh* mutant, but not the Δ*ldhA* and Δ*mdh* strains, showed reduced growth and a lowered NAD^+^/NADH ratio, in line with Ndh being the major enzyme for NADH oxidation. Growth of the double mutants Δ*ndh*Δ*mdh* and Δ*ndh*Δ*ldhA*, but not of strain Δ*mdh*Δ*ldhA*, in glucose medium was stronger impaired than that of the Δ*ndh* mutant, supporting an active role of the alternative Mdh-Mqo and LdhA-LldD systems in NADH oxidation and menaquinone reduction. In L-lactate minimal medium the Δ*ndh* mutant grew better than the wild type, probably due to a higher activity of the menaquinone-dependent L-lactate dehydrogenase LldD. The Δ*ndh*Δ*mdh* mutant failed to grow in L-lactate medium and acetate medium. Growth with L-lactate could be restored by additional deletion of *sugR*, suggesting that *ldhA* repression by the transcriptional regulator SugR prevented growth on L-lactate medium. Attempts to construct a Δ*ndh*Δ*mdh*Δ*ldhA* triple mutant were not successful, suggesting that Ndh, Mdh and LdhA cannot be replaced by other NADH-oxidizing enzymes in *C. glutamicum*.

## Introduction

*Corynebacterium glutamicum* is a Gram-positive, non-pathogenic soil bacterium that was isolated in a screen for microorganisms which excrete the flavor enhancer L-glutamate (Kinoshita et al., 1957). It is used in industry for the biotechnological production of amino acids and proteins (Eggeling and Bott, 2015; Freudl, 2017). Furthermore, strains for efficient production of many other metabolites have been constructed, such as organic acids (Wieschalka et al., 2013), diamines (Wendisch et al., 2018), and various other compounds (Becker and Wittmann, 2012). The majority of the corresponding production processes are performed under aerobic conditions and require a functional respiratory chain (Bott and Niebisch, 2003; Matsushita, 2013). Several dehydrogenases oxidize their substrates with concomitant reduction of menaquinone, the only respiratory quinone in corynebacteria. Finally, electron transfer from menaquinol to oxygen is catalyzed either by a cytochrome *bc*_1_*-aa*_3_ supercomplex (Niebisch and Bott, 2003; Graf et al., 2016; Kao et al., 2016) or by cytochrome *bd* oxidase (Kusumoto et al., 2000; Kabus et al., 2007).

**Figure 1:**
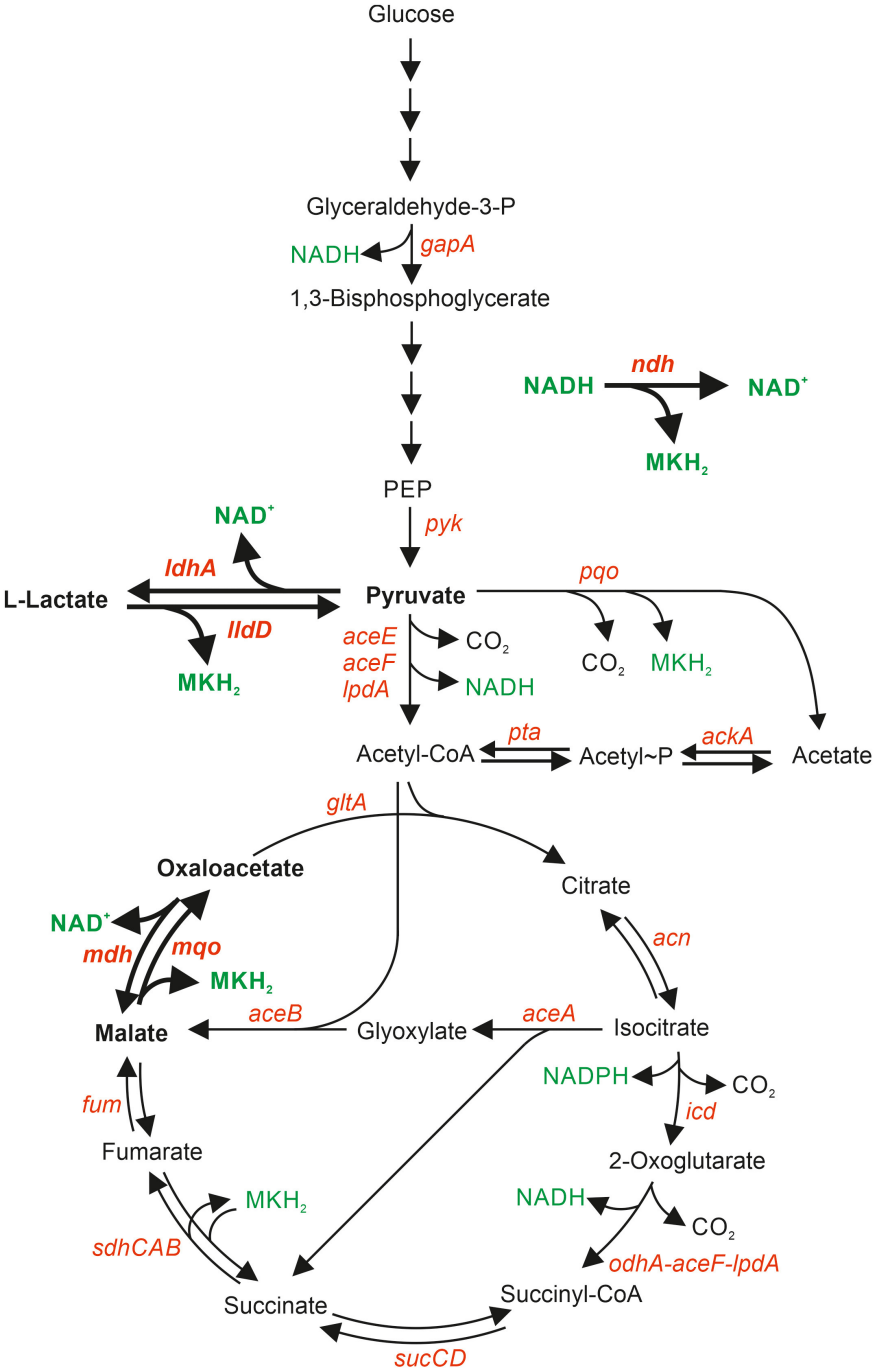
Central metabolic pathways relevant for NADH formation and oxidation in *C. glutamicum*. Genes are shown in red, NAD^+^, NADH, NADPH, and MKH_2_ in green. Reactions, metabolites, and genes relevant for NADH oxidation coupled to menaquinone (MK) reduction are shown in bold.

As the majority of reducing equivalents from substrate oxidation are initially transferred to NAD^+^, a sufficient rate of NADH reoxidation is crucial to sustain carbon flux in central metabolism. *C. glutamicum* possesses a non-proton-pumping, single-subunit NADH dehydrogenase Ndh (Nantapong et al., 2005), but neither a proton-pumping complex I-type NADH dehydrogenase (Parey et al., 2020) nor a sodium-ion-pumping Nqr-type NADH dehydrogenase (Steuber et al., 2014). Analysis of a Δ*ndh* mutant of *C. glutamicum* revealed slightly decreased growth on glucose and acetate minimal agar plates and almost no growth defect in glucose minimal medium (Molenaar et al., 2000; Nantapong et al., 2004). Two alternative systems were proposed that can couple the oxidation of NADH to menaquinone reduction and thus compensate the lack of Ndh (Molenaar et al., 2000; Nantapong et al., 2004). These are the NAD^+^-dependent malate dehydrogenase Mdh (Genda et al., 2003) acting in concert with the membrane-associated malate:quinone oxidodreductase Mqo, and the NAD^+^-dependent L-lactate dehydrogenase LdhA acting in concert with the membrane-associated L-lactate dehydrogenase LldD (Figure 1).

The net reaction of the Mdh-Mqo couple equals that of an Ndh and it could serve as an alternative “NADH dehydrogenase,” as Mdh reduces oxaloacetate with NADH to L-malate (ΔG^0′^ = −28.9 kJ/mol), and the membrane-associated Mqo subsequently re-oxidizes L-malate back to oxaloacetate and reduces MK (ΔG^0′^ = −18.5 kJ/mol). It was shown that Mqo is crucial for oxidation of malate to oxaloacetate during aerobic growth of *C. glutamicum* (Molenaar et al., 2000). Mdh was able to complement the absence of Mqo activity only when the Δ*mqo* mutant was supplied with nicotinamide in order to increase the concentrations of NAD^+^ and NADH (Molenaar et al., 2000). Under these conditions, the NAD^+^/NADH ratio decreased from 4.4 in the absence of nicotinamide to 2.8 in the presence of 1 mg L^−^^1^ nicotinamide, which presumably enabled the thermodynamically unfavorable oxidation of malate by Mdh (Molenaar et al., 2000). Similar to the Mdh-Mqo couple, the net reaction of the LdhA-LldD couple also corresponds to that of Ndh, as LdhA reduces pyruvate with NADH to L-lactate (ΔG^0′^ = −25.1 kJ/mol) and LldD re-oxidizes L-lactate to pyruvate and reduces MK (ΔG^0′^ = −22.4 kJ/mol) (Nantapong et al., 2004).

The Mdh-Mqo and LdhA-LldD couples might function as overflow systems that come into play when Ndh activity is insufficient. For example, an F_1_F_O_-ATP synthase mutant with 25% residual ATPase activity showed enhanced glucose consumption and respiration rates (Sekine et al., 2001), requiring increased NADH oxidation rates. A proteome comparison of this ATPase mutant with the wild type revealed that the amounts of Mqo and Mdh were increased (Li et al., 2007). The authors suggested that the Mdh-Mqo couple enabled the increased NADH oxidation rates in the ATP synthase mutant (Sawada et al., 2012). A ΔF_1_F_O_ mutant of *C. glutamicum* completely devoid of ΔF_1_F_O_-ATP synthase activity also showed increased glucose consumption and respiration rates and both the mRNA level and the protein level of Mdh were increased (Koch-Koerfges et al., 2012).

In this study, we aimed at getting a better understanding of the role of the different NADH oxidation systems for growth and metabolism of *C. glutamicum*. For this purpose, we constructed and characterized six deletion mutants that lack one or two of the NADH oxidation systems. The phenotype of the different mutant strains strongly depended on the carbon source. In our study, we tested glucose, acetate, and L-lactate. The results indicate that NADH oxidation is predominantly catalyzed by Ndh, but that the Mdh-Mqo and LdhA-LldD couples are also involved and increase metabolic flexibility.

## Materials and Methods

### Bacterial Strains and Culture Conditions

*C. glutamicum* strains and plasmids used in this work are listed in Table 1. For the analysis of growth, organic acid production, carbon source consumption, oxygen consumption, enzyme activities, and NAD^+^/NADH ratios, a 5 ml preculture in brain-heart infusion (BHI) medium was inoculated with colonies from a fresh agar plate (BHI agar) and incubated for 8 h at 30°C and 170 rpm. Cells from the preculture were transferred into 20 ml CGXII minimal medium (Keilhauer et al., 1993) containing either 4% (wt/vol) glucose (1,071 mM C), or 2–4% (wt/vol) sodium L-lactate (536–1,071 mM C), or 2–4% (wt/vol) sodium acetate (488–975 mM C) as carbon and energy source. The cultures were incubated for 16 h at 30°C and 130 rpm. After washing the cells with 0.9% (wt/vol) NaCl, the main culture with 50 ml CGXII minimal medium containing 4% (wt/vol) of the desired carbon source was inoculated to give an optical density at 600 nm (OD_600_) of 1. The CGXII medium was always supplemented with 30 mg/l 3,4-dihydroxybenzoic acid as iron chelator (Frunzke et al., 2008). The main cultivations were performed in baffled 500 ml Erlenmeyer flasks containing a septum for sterile sampling of the cultures at 30°C and 130 rpm. Cells were harvested during the exponential growth phase at an OD_600_ of 4–6 for further analysis. *E. coli* DH5α, which was used as host for cloning, was cultivated in LB medium or on LB agar plates at 37°C. When appropriate, kanamycin was used at a concentration of 25 μg/ml (*C. glutamicum*) or 50 μg/ml (*E. coli*).

**Table 1:** Strains and plasmids used in this study.

**Strain or plasmid**	**Relevant characteristic(s)**	**Source or references**
**STRAINS**
*C. glutamicum* ATCC 13032	Biotin-auxotrophic wild type	Kinoshita et al., 1957
Δ*ldhA*	13032 derivative with an in-frame deletion of *ldhA* (cg3219)	This study
Δ*mdh*	13032 derivative with an in-frame deletion of *mdh* (cg2613)	This study
Δ*ndh*	13032 derivative with an in-frame deletion of *ndh* (cg1656)	This study
Δ*mdh*Δ*ldhA*	13032 derivative with in-frame deletions of *mdh* and *ldhA*	This study
Δ*ndh*Δ*mdh*	13032 derivative with in-frame deletions of *ndh* and *mdh*	This study
Δ*ndh*Δ*ldhA*	13032 derivative with in-frame deletions of *ndh* and *ldhA*	This study
Δ*ndh*Δ*mdh*Δ*sugR*	13032 derivative with in-frame deletions of *ndh, mdh, and sugR* (cg2115)	This study
*E. coli* DH5α	F^−^Φ80*dlac*Δ(*lacZ*)M15 Δ(*lacZYA*-*argF*) U169 *endA1recA1 hsdR17* (r_K_−, m_K_+) *deoR thi-1 phoA supE44*λ^−^*gyrA96 relA1*	Invitrogen (Karlsruhe, Germany)
**PLASMIDS**
pK19*mobsacB*	Km^r^, *oriV_*E*.*coli*_ oriT sacB* integration vector for allelic exchange in *C. glutamicum*	Schäfer et al., 1994
pK19*mobsacB*-Δ*ldhA*	Kan^R^, pK19*mobsacB* derivative containing an overlap- extension PCR product covering the up- and downstream regions of *ldhA*	Litsanov et al., 2012
pK19*mobsacB*-Δ*mdh*	Kan^R^, pK19*mobsacB* derivative containing an overlap- extension PCR product covering the up- and downstream regions of *mdh*	This study
pK19*mobsacB*-Δ*ndh*	Kan^R^, pK19*mobsacB* derivative containing an overlap- extension PCR product covering the up- and downstream regions of *ndh*	This study
pK19*mobsacB*-Δ*sugR*	Kan^R^, pK19*mobsacB* derivative containing an overlap- extension PCR product covering the up- and downstream regions of *sugR*	Engels and Wendisch, 2007
pAN6	Km^R^; *C. glutamicum/E. coli* shuttle vector for regulated gene expression (P*_*tac*_, lacI*^q^, pBL1 *oriV_*C*.*glutamicum*_*, pUC18 *oriV_*E*.*coli*_*)	Frunzke et al., 2008
pAN6-*ldhA*	Km^R^; pAN6 derivative containing the gene *ldhA*	This study
pAN6-*mdh*	Km^R^; pAN6 derivative containing the gene *mdh*	This study
pAN6-*ndh*	Km^R^; pAN6 derivative containing the gene *ndh*	This study

### Construction of pK19*mobsacB* Plasmids and Deletion Mutants

In-frame deletion mutants of C. glutamicum ATCC 13032 were constructed as described previously (Niebisch and Bott, 2001). For this purpose, the *ndh* or *mdh* upstream region, including the first seven to ten codons of each gene, and the downstream region, including the last seven codons of each gene, were amplified with the Expand High Fidelity kit (Roche Diagnostics, Mannheim, Germany) using the oligonucleotide pairs ΔXXX-1-for/ΔXXX-2-rev and ΔXXX-3-for/ΔXXX-4-rev, respectively. The “XXX” stands for the name of the gene to be deleted and is specified in Table 2, which lists the oligonucleotides used in this study. The resulting PCR products of about 500 bp were subsequently fused by overlap-extension PCR to products of ~1,000 bp. After digestion with *Xma*I and *Xba*I, these fragments were cloned into pK19*mobsacB* (Schäfer et al., 1994), cut with the same restriction enzymes, to yield pK19*mobsacB-*Δ*ndh* and pK19*mobsacB-*Δ*mdh*, respectively. DNA sequence analysis revealed that the cloned PCR products contained no unwanted mutations. Subsequently, the plasmids were transferred by electroporation (van der Rest et al., 1999) into *C. glutamicum* strain ATCC 13032 and the transformation mixture was plated on a BHIS agar plate containing 25 μg kanamycin/ml. After selection for the first and second recombination event, kanamycin-sensitive and sucrose-resistant clones were analyzed by colony-PCR with the primer pair ΔXXX-fw/ΔXXX-rev in order to distinguish between wild type and Δ*ndh* or Δ*mdh* clones, respectively. For the construction of the double deletion strains lacking two NADH oxidation systems, namely Δ*ndh*Δ*mdh*, Δ*ndh*Δ*ldhA*, and Δ*mdh*Δ*ldhA*, pK19mobsacB-Δ*mdh* or pK19*mobsacB*-Δ*ldhA* were transferred by electroporation into competent Δ*ndh* or Δ*mdh* cells and selected and analyzed as described above. For the construction of the triple deletion strain Δ*ndh*Δ*mdh*Δ*sugR*, pK19*mobsacB*-Δ*sugR* was transferred by electroporation into competent Δ*ndh*Δ*mdh* cells and selected and analyzed as described above.

**Table 2:** Oligonucleotides used in this study.

**Oligonucleotide**	**Sequence**	**Application**
Δ*mdh*-1-for	5′-TAATCTAGACGCTTGGACATGCCAGATGCCTT-3′	Amplification of *mdh* upstream region, includes XmaI restriction site
Δ*mdh*-2-rev	5′*CCCCGTAACTAAACTTAAACA*GACGTTCTGCGGGGAATTCAT-3′	Amplification of *mdh* upstream region
Δ*mdh*-3-for	5′-*TGTTTAAGTTTAGTTACGGGG*GCAGTGCGCGACTTGCTCTAA-3′	Amplification of *mdh* downstream region
Δ*mdh*-4-rev	5′-TAACCCGGGGCTTGATAAATCCACGCTGGG-3′	Amplification of *mdh* downstream region, includes XbaI restriction site
Δ*mdh*-fw	5′-CCTTTCTTATCGCCAAAGTGA-3′	Control of *mdh* deletion *via* colony PCR
Δ*mdh*-rev	5′-GCGGGTCGGATTCCACG-3′	Control of *mdh* deletion *via* colony PCR
*mdh*-F	5′-TGGCATATGAATTCCCCGCAGAACGTC-3′	Amplification of *mdh* for expression plasmid pAN6-*mdh*, includes NdeI restriction site
*mdh*-R	5′-GTGCTAGCTTAGAGCAAGTCGCGCACTGCC-3′	Amplification of *mdh* for expression plasmid pAN6-*mdh*, includes NheI restriction site
Δ*ndh*-1-for	5′-TAATCTAGAACCCCAGGCCACTCTTCC-3′	Amplification of *ndh* upstream region, includes XmaI restriction site
Δ*ndh*-2-rev	5′-*CCCCATCCACTAAACTTAAACA*GCCTTCGGGGCGGGTTGGGTT-3′	Amplification of *ndh* upstream region
Δ*ndh*-3-for	5′-*TGTTTAAGTTTAGTTACGGGG*CAGCGTTTCAGCGGAAAGTAA-3′	Amplification of *ndh* downstream region
Δ*ndh*-4-rev	5′-TAACCCGGGCAGAAGGAGTTCCCGCATTGA-3′	Amplification of *ndh* downstream region, includes XbaI restriction site
Δ*ndh*-fw	5′-CCAGCAAACGCTAGGTTGGG-3′	Control of *ndh* deletion *via* colony PCR
Δ*ndh*-rev	5′-TGCACCCTCAATGGCCTTGGT-3′	Control of *ndh* deletion *via* colony PCR
*ndh*-F	5′-GCGCATATGTCAGTTAACCCAACCC-3′	Amplification of *ndh* for expression plasmid pAN6-*ndh*, includes NdeI restriction site
*ndh*-R	5′-GCGCTAGCTTACTTTCCGCTGAAACG-3′	Amplification of *ndh* for expression plasmid pAN6-*ndh*, includes NheI restriction site
Δ*ldhA*-fw	5′-GCACCAGTTGCGATGTGGGTGG-3′	Control of *ldhA* deletion *via* colony PCR
Δ*ldhA*-rev	5′-CGTTGTCGATCATCTGCTTCCAG-3′	Control of *ldhA* deletion *via* colony PCR
*ldhA*-F	5′-AAACATATGAAAGAAACCGTCGGTAACAAGATTG-3′	Amplification of *ldhA* for expression plasmid pAN6-*ldhA*, includes NdeI restriction site
*ldhA*-R	5′-AAAGCTAGCTTAGAAGAACTGCTTCTGAATTTCGCGCAG-3′	Amplification of *ldhA* for expression plasmid pAN6-*ldhA*, includes NheI restriction site

*Restriction sites are underlined, other functional regions are italic*.

### Construction of pAN6-Based Expression Plasmids

The genes *ldhA, mdh*, and *ndh* were amplified from chromosomal DNA of *C. glutamicum* ATCC 13032 with the gene-specific oligonucleotides XXX_F and XXX_R (Table 2). The former one introduced an *Nde*I restriction site including the start codon, the latter one an *Nhe*I restriction site. The resulting PCR products, with a size of 960 bp for *ldhA*, 1,001 bp for *mdh*, and 1,418 bp for *ndh*, were cut with *Nde*I and *Nhe*I and cloned separately into the expression plasmid pAN6 (Frunzke et al., 2008) cut with the same enzymes. The correctness of the cloned DNA fragments was confirmed by DNA sequencing. Subsequently, the plasmids were transferred by electroporation into *C. glutamicum* wild type and mutants and the transformation mixture was plated on a BHIS agar plate containing 25 μg/mL^−^^1^ kanamycin. Enzymes used for DNA restriction, ligation or dephosphorylation were obtained either from Roche Diagnostics (Mannheim, Germany) or New England Biolabs (Frankfurt am Main, Germany). Plasmid DNA from *E. coli* or *C. glutamicum* was isolated with the QIAprep Spin Miniprep kit according to the manufacturer's instructions (Qiagen, Hilden, Germany).

### Determination of Growth Parameters and Consumption Rates of Glucose, Organic Acids, and Oxygen

Growth was followed by measuring the optical density at 600 nm (OD_600_) with an Ultrospec 500-pro spectrophotometer (Amersham Biotech). The biomass concentration was calculated from OD_600_ values using an experimentally determined correlation factor of 0.25 g_CDW_ L^−^^1^ for OD_600_ = 1 (Kabus et al., 2007). Quantitative determination of glucose and organic acids in culture supernatants, and calculation of carbon source uptake rates was carried out as described (Koch-Koerfges et al., 2012).

Oxygen uptake rates (OUR) of non-growing cells were measured with a Clarke-type oxygen electrode using a thermostatically controlled, magnetically stirred 2-ml chamber at 30°C (Oxygraph, Hansatech Instruments, Germany) as described previously (Koch-Koerfges et al., 2013). The cells where harvested during exponential growth in shake flasks and oxygen consumption was followed using a cell suspension with an OD_600_ of 0.5–2.5 in the chamber. The protein content was calculated by assuming that 50% of the cell dry weight corresponds to protein.

### Preparation of Cell Fractions for Enzymatic Assays

*C. glutamicum* cells were harvested by centrifugation (10,000 *g*, 4°C, 5 min) during the exponential growth phase and washed twice with 20 mM potassium phosphate buffer (PPB), pH 7.5. One ml of the cell suspension was mixed with 1 g zirconia/silica beads (0.1 mm diameter; Biospec, Bartlesville, USA) in a 2-ml Eppendorf tube and the cells were mechanically disrupted by three 30 s shakings in a Silamat S5 (Ivoclar Vivadent, Ellwangen, Germany). Cell debris and unbroken cells were separated by centrifugation for 5 min at 16,000 *g* and 4°C. The supernatant was then ultracentrifuged at 171,000 *g* for 60 min at 4°C. The resulting supernatant containing the soluble proteins and the resuspended sedimented membrane fraction (in 20 mM PPB pH 7.5) were used for the assays.

### Enzyme Activity of Membrane-Bound Enzymes

LldD and Mqo activities were measured spectrophotometrically at 25°C by following the change in the absorbance of 2,6-dichlorophenolindophenol (DCPIP) at 600 nm (Molenaar et al., 2000; Nantapong et al., 2004). The reaction mixture contained 100 μg membrane protein, 0.2 mM DCPIP, 0.4 mM phenazine methosulfate (PMS), 1 mM NaN_3_, 1 mM substrate (L-lactate or L-malate for LldD and Mqo, respectively), and 50 mM PPB, pH 7.0, in a total volume of 1 ml. The amount of enzyme reducing 1 μmol/min DCPIP was defined as 1 unit, using an extinction coefficient of 22 mM^−1^ cm^−1^. Ndh activity was determined as described previously (Molenaar et al., 2000). The reaction mixture contained 100 μg membrane fraction, 0.4 mM DCPIP, 1 mM NaN_3_, 0.4 mM NADH, and 50 mM PPB, pH 6.5, in a total volume of 1 ml. Before adding the substrate, the reaction mixtures were incubated for 5 min at 25°C. Using a reaction mixture with buffer instead of membrane protein as a control, the decrease in absorption at 340 nm was measured and subtracted from each measurement to calculate the net value. The activity unit was defined as 1 μmol NADH oxidized per min, using an extinction coefficient of 6.3 mM^−1^ cm^−1^.

### Enzyme Activity of Cytoplasmic Enzymes

LdhA and Mdh activities were determined spectrophotometrically at 25°C by following the decrease in the absorbance of NADH at 340 nm. The reaction mixture contained 100 μg soluble protein fraction, 0.2 mM NADH, 1 mM pyruvate or oxaloacetate (for Ldh or Mdh, respectively), and 50 mM PPB (pH 7.0 or pH 7.5, for the Ldh or Mdh activity assay, respectively) in a total volume of 1 ml. The activity unit was defined as 1 μmol/min NADH oxidized, using an extinction coefficient of 6.3 mM^−1^ cm^−1^. All enzyme activity results for cells grown on glucose, acetate or L-lactate minimal medium were performed in triplicates.

### Determination of Intracellular NAD^+^/NADH Ratios

The concentrations of the pyridine nucleotides NADH and NAD^+^ were determined with the EnzyChrom™ NAD^+^/NADH assay kit (BioAssay Systems, Hayward, USA) following the manufacturer's instructions and as described previously (Siedler et al., 2011). Cells in the exponential growth phase were cooled with ice water, harvested by centrifugation (10,000 *g*, 4°C, 5 min), and washed twice with cold phosphate-buffered saline (PBS), pH 7.5. The sedimented cells were resuspended either in acid extraction buffer or in base extraction buffer (both part of the kit) to extract oxidized pyridine nucleotides or reduced pyridine nucleotides, respectively. The concentrations of NAD^+^ and NADH in each extract were then quantified by the lactate dehydrogenase cycling reaction, in which the formed NADH reduces a formazan (MTT) reagent.

## Results

### Growth of the *C. glutamicum* Strains Δ*ndh*, Δ*mdh*, and Δ*ldhA* on Glucose and Acetate

In order to determine the relevance of NADH dehydrogenase Ndh, malate dehydrogenase Mdh and L-lactate dehydrogenase LdhA for aerobic growth of *C. glutamicum*, we characterized the deletion mutants Δ*ndh*, Δ*mdh*, and Δ*ldhA* during cultivation in shake flasks with glucose, L-lactate, or acetate, as carbon source. These substrates were chosen as their oxidation is linked to different numbers of NADH-forming reactions. Glucose oxidation to 6 CO_2_ involves three NADH-forming reactions [glyceraldehyde 3-phosphate dehydrogenase, pyruvate dehydrogenase complex, and unusual 2-oxoglutarate dehydrogenase complex (Niebisch et al., 2006; Hoffelder et al., 2010; Bruch et al., 2020; Kinugawa et al., 2020)] and is coupled to the formation of 6 NADH, 2 NADPH, and 4 MQH_2_ if the oxidative pentose phosphate pathway is neglected. l-Lactate oxidation to 3 CO_2_ involves two NADH-forming reactions (pyruvate dehydrogenase complex, and 2-oxoglutarate dehydrogenase complex) and leads to the formation of 2 NADH, 1 NADPH, and 3 MQH_2_. Acetate oxidation to 2 CO_2_ involves only one NADH-forming reaction (2-oxoglutarate dehydrogenase complex) and is linked to the formation of 1 NADH, 1 NADPH, and 2 MQH_2_ (Figure 1).

**Figure 2:**
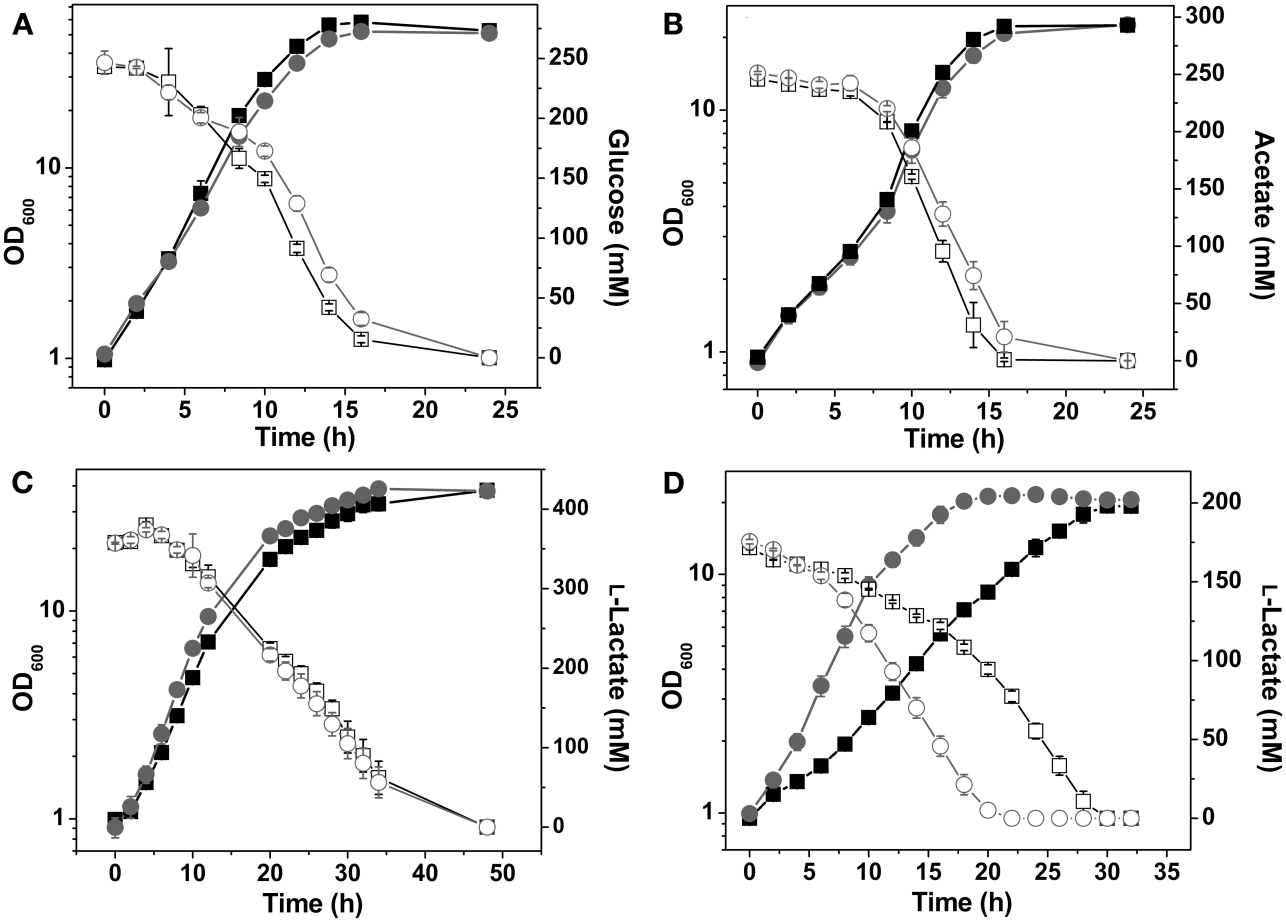
Growth and carbon source consumption of *C. glutamicum* wild type (■) and the Δ*ndh* mutant (●). Cells were grown in CGXII minimal medium containing 4% (wt/vol) glucose **(A)**, 2% (wt/vol) acetate **(B)**, 4% (wt/vol) L-lactate **(C)**, or 2% (wt/vol) L-lactate **(D)**. In **(C)** cells were precultivated in L-lactate minimal medium, in **(D)** cells were precultivated in glucose minimal medium. The concentrations of the individual carbon sources in the culture supernatant are shown by open squares (□) for the wild type and by open circles (○) for the Δ*ndh* mutant. Average values from at least three independent cultivations and standard deviations are shown.

In glucose minimal medium the Δ*ndh* mutant showed an 18% decreased growth rate and a 12% reduced biomass formation compared to the wild type (Figure 2A, Table 3). NADH dehydrogenase activity was not detectable in extracts of the Δ*ndh* mutant, whereas it was high (2.4 U mg^−1^) in the wild type (Table 4). The Δ*ndh* mutant showed a 28% decreased glucose uptake rate (GUR, Table 3) and a 27% decreased oxygen uptake rate compared to the wild type (OUR, 185 ± 13 vs. 254 ± 6 nmol O_2_ mg_Protein_−1 min^−1^), suggesting that glycolytic flux and consequently also respiration were reduced due to an insufficient rate of NADH oxidation in the absence of Ndh. In agreement with this assumption, the NAD^+^/NADH ratio was reduced by more than 50% in the Δ*ndh* mutant compared to the wild type (2.0 vs. 4.7). Furthermore, the Δ*ndh* mutant secreted 22% more lactate than the wild type (Table 3). Pyruvate reduction to lactate by LdhA represents an alternative path for NADH oxidation. The enzyme activities of LdhA, Mdh, and Mqo were unchanged in the Δ*ndh* mutant, whereas LldD activity was increased 3.3-fold (Table 4).

**Table 3:** Growth rate, biomass formation (max. OD600), carbon source uptake rates, and maximal organic acid formation of the *C. glutamicum* wild type, and its Δ*ndh*, Δ*mdh*Δ*ldhA*, Δ*ndh*Δ*mdh* and Δ*ndh*Δ*ldhA* mutants.

**Carbon source**	**Strain**	**μ (h^**−1**^)**	**Max. OD_**600**_**	**Carbon source uptake rates (mmol min^−1^ mg_CDW_−1)**	**Maximal organic acid formation (mM)**			
					**L-Lactate**	**Acetate**	**Succinate**	**Pyruvate**
Glucose	WT	0.39 ± 0.01	62.2 ± 0.9	88 ± 9 (528 ± 54)^a^	96 ± 5	24 ± 2	4.3 ± 0.5	0.4 ± 0.4
	Δ*ndh*	0.32 ± 0.01	50.9 ± 1.5	64 ± 2 (384 ± 12)	117 ± 4	28 ± 11	1.9 ± 0.3	0.5 ± 0.3
	Δ*mdh*Δ*ldhA*	0.36 ± 0.01	49.3 ± 0.9	76 ± 4 (456 ± 24)	N.D.^b^	12 ± 1.8	6.4 ± 2.8	4.1 ± 0.3
	Δ*ndh*Δ*ldhA*	0.25 ± 0.01	37.4 ± 2.6	53 ± 2 (318 ± 12)	N.D.	85 ± 14	14 ± 3	11 ± 1
	Δ*ndh*Δ*mdh*	0.24 ± 0.01	52.9 ± 0.4	59 ± 3 (354 ± 18)	121 ± 3	5.7 ± 0.1	1.4 ± 0.3	2.5 ± 1.1
L-Lactate	WT	0.19 ± 0.01	36.6 ± 0.2	98 ± 5 (294 ± 15)	–	N.D.	0.5 ± 0.1	3.9 ± 0.7
	Δ*ndh*	0.22 ± 0.01	38.2 ± 0.2	115 ± 5 (345 ± 15)	–	N.D.	N.D.	0.8 ± 0.2
	Δ*mdh*Δ*ldhA*	0.19 ± 0.02	36.7 ± 1.0	105 ± 8 (315 ± 24)	–	N.D.	1.0 ± 0.2	2.7 ± 0.3
	Δ*ndh*Δ*ldhA*	0.18 ± 0.01	36.0 ± 0.4	99 ± 3 (297 ± 9)	–	N.D.	N.D.	15 ± 1
	Δ*ndh*Δ*mdh*	N.G.^c^; M.A.^d^	–	95 ± 4 (285 ± 12)	–	N.D.	N.D.	21 ± 2
Acetate	WT	0.29 ± 0.01	23.1 ± 0.8	231 ± 4 (462 ± 8)	0.8 ± 0.1	–	N.D.	N.D.
	Δ*ndh*	0.27 ± 0.01	23.5 ± 0.4	200 ± 10 (400 ± 20)	0.8 ± 0.1	–	N.D.	N.D.
	Δ*mdh*Δ*ldhA*	0.28 ± 0.01	21.3 ± 0.5	230 ± 11 (460 ± 22)	N.D.	–	N.D.	N.D.
	Δ*ndh*Δ*ldhA*	0.26 ± 0.01	22.9 ± 0.3	192 ± 3 (384 ± 6)	N.D.	–	N.D.	N.D.
	Δ*ndh*Δ*mdh*	N.G.	–	–	–	–	–	–

*Cells were cultivated in shake flasks as described in Materials and Methods. Mean values and standard deviations from three independent cultivations are shown*.

a *Numbers in parenthesis give uptake rate as mmol C min^−1^ mg_CDW_−1*.

b *N.D., not detected*.

c *N.G., no growth*.

d *M.A., metabolically active*.

**Table 4:** NAD^+^/NADH ratios and enzyme activities in *C. glutamicum* wild type and its Δ*ndh*, Δ*mdh*Δ*ldhA*, Δ*ndh*Δ*ldhA*, and Δ*ndh*Δ*mdh* mutants.

**Carbon source**	**Strain**	**NAD^**+**^/NADH ratio**	**Enzyme activity (Units/mg**_****Protein****_**)**				
			**LdhA**	**LldD**	**Mdh**	**Mqo**	**Ndh**
Glucose	WT	4.7 ± 0.3	0.35 ± 0.01	0.03 ± 0.01	2.02 ± 0.24	0.10 ± 0.03	2.37 ± 0.29
	Δ*ndh*	2.0 ± 0.1	0.37 ± 0.01	0.10 ± 0.02	1.93 ± 0.11	0.10 ± 0.02	N.D.^a^
	Δ*mdh*	4.7 ± 0.1	0.35 ± 0.01	0.02 ± <0.01	0.01 ± <0.01	0.10 ± 0.02	2.10 ± 0.10
	Δ*ldhA*	5.4 ± 0.3	0.01 ± <0.01	0.01 ± <0.01	2.06 ± 0.12	0.13 ± 0.01	2.32 ± 0.21
	Δ*mdh*Δ*ldhA*	2.5 ± 0.2	0.01 ± 0.01	0.01 ± 0.01	0.01 ± 0.01	0.08 ± 0.04	2.46 ± 0.13
	Δ*ndh*Δ*ldhA*	1.8 ± 0.1	0.01 ± 0.01	0.01 ± 0.01	2.09 ± 0.20	0.10 ± 0.01	N.D.
	Δ*ndh*Δ*mdh*	1.3 ± 0.1	0.40 ± 0.01	0.31 ± 0.04	0.02 ± 0.01	0.14 ± 0.01	N.D.
L-Lactate	WT	2.8 ± 0.1	0.03 ± 0.01	0.13 ± 0.01	3.00 ± 0.26	0.12 ± 0.05	0.93 ± 0.17
	Δ*ndh*	2.1 ± 0.1	0.03 ± 0.01	0.21 ± 0.01	2.80 ± 0.27	0.11 ± 0.02	N.D.
	Δ*mdh*Δ*ldhA*	2.4 ± 0.1	0.01 ± 0.01	0.17 ± 0.01	0.01 ± 0.01	0.10 ± 0.02	0.96 ± 0.31
	Δ*ndh*Δ*ldhA*	2.2 ± 0.1	0.01 ± 0.01	0.15 ± 0.01	3.04 ± 0.22	0.12 ± 0.01	N.D.
	Δ*ndh*Δ*mdh*	N.G.^b^	N.G.	N.G.	N.G.	N.G.	N.G.
Acetate	WT	2.8 ± 0.1	0.03 ± 0.01	0.03 ± 0.01	2.69 ± 0.13	0.29 ± 0.02	1.41 ± 0.18
	Δ*ndh*	2.3 ± 0.3	0.03 ± 0.01	0.03 ± 0.01	2.10 ± 0.08	0.23 ± 0.03	N.D.
	Δ*mdh*Δ*ldhA*	2.4 ± 0.2	0.01 ± 0.01	0.01 ± 0.01	0.01 ± 0.01	0.24 ± 0.01	1.21 ± 0.27
	Δ*ndh*Δ*ldhA*	2.1 ± 0.3	0.01 ± 0.01	0.01 ± 0.01	2.88 ± 0.16	0.25 ± 0.03	N.D.
	Δ*ndh*Δ*mdh*	N.G.	N.G.	N.G.	N.G.	N.G.	N.G.

*Cells were cultivated in shake flasks as described in Materials and Methods and harvested in the early exponential growth phase before the onset of oxygen limitation. Mean values and standard deviations from three independent cultivations are shown*.

a *N.D., not detected*.

b *N.G., no growth*.

In acetate minimal medium, the growth rate of the Δ*ndh* mutant was slightly decreased by 7% (Figure 2B), the acetate uptake rate (AUR) was reduced by 13%, and the biomass yield was unchanged compared to the wild type (Table 3). The Mdh and Mqo activities were reduced by about 20% (Table 4). The NAD^+^/NADH ratio during growth on acetate was reduced by 18% in the Δ*ndh* mutant compared to the wild type (2.3 vs. 2.8 in the wild type, Table 4). It is noticeable that the NAD^+^/NADH ratio of the wild type on acetate was 40% lower than on glucose.

In contrast to the Δ*ndh* strain, growth of the Δ*mdh* and Δ*ldhA* mutants on glucose or acetate was comparable to that of the wild type and no changes of the NAD^+^/NADH ratio and of any enzyme activity tested except for the one deleted were observed (data not shown).

### Growth of the Δ*ndh* Mutant on L-lactate

When cultivated in CGXII medium with L-lactate as sole carbon source, the Δ*ndh* mutant grew slightly faster than the wild type (μ = 0.22 vs. 0.19 h^−1^), whereas biomass formation was unchanged (Figure 2C). While the wild type required pre-cultivation in L-lactate medium to reach optimal growth rates on L-lactate, the Δ*ndh* mutant showed optimal growth irrespective of the carbon source used in the preculture (Figures 2C,D). The growth rate of non-adapted wild-type cells on L-lactate (μ = 0.13 h^−1^) was 34% lower compared to that of L-lactate-adapted wild-type cells (μ = 0.19 h^−1^). The improved growth of the Δ*ndh* mutant on L-lactate could be reversed by expression of *ndh* using the expression plasmid pAN6-*ndh* (data not shown). This indicates that the absence of Ndh has a positive influence on l-lactate utilization. The NAD^+^/NADH ratio on lactate was 2.8 for the wild type, comparable to acetate-grown cells, and 2.1 for the Δ*ndh* mutant. Similar to glucose-grown cells, the LldD activity was 1.6-fold increased in a Δ*ndh* mutant grown in l-lactate medium and the oxygen consumption rate of the Δ*ndh* mutant with l-lactate as substrate was increased by 17% compared to the wild type (335 ± 17 vs. 285 ± 15 nmol O_2_ mg_Protein_−1 min^−1^). The increased LldD activity of the Δ*ndh* mutant is presumably responsible for the increased growth rate and the increased oxygen consumption rate. The wild type secreted up to 3.9 mM pyruvate during the early exponential phase, which was metabolized during mid and late exponential phase, whereas the Δ*ndh* mutant secreted below 1 mM pyruvate (Table 3).

### Characteristics of a Δ*mdh*Δ*ldhA* Double Mutant

To explore how the absence of more than one NADH oxidation system influences growth and other parameters, we constructed the *C. glutamicum* strains Δ*mdh*Δ*ldhA*, Δ*ndh*Δ*ldhA*, and Δ*ndh*Δ*mdh*. Attempts to construct the triple mutant Δ*ndh*Δ*mdh*Δ*ldhA* failed, suggesting that under the conditions tested no further efficient NADH oxidation system exists in *C. glutamicum* that can compensate the lack of Ndh, LdhA, and Mdh.

**Figure 3:**
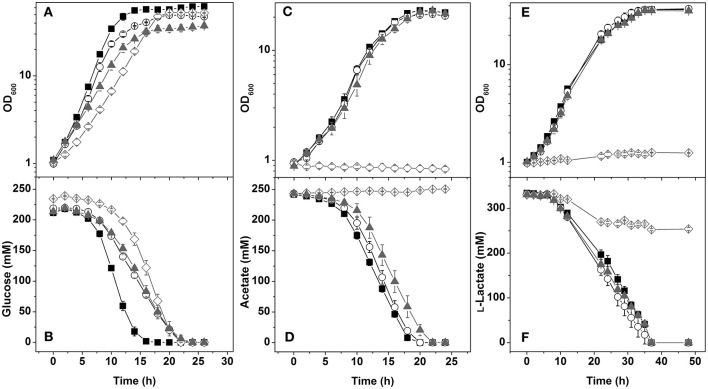
Growth and carbon source consumption of the *C. glutamicum* wild type (■) and the mutants Δ*mdh*Δ*ldhA* (○), Δ*ndh*Δ*ldhA* (▴), and Δ*ndh*Δ*mdh* (♢). Cells were cultivated in CGXII minimal medium containing either 4% (wt/vol) glucose **(A,B)**, 2% (wt/vol) acetate **(C,D)**, or 4% (wt/vol) l-lactate **(E,F)**. Growth **(A,C,E)** and carbon source concentration in the supernatants **(B,D,F)** are shown. Average values from at least three independent cultivations and the standard deviations are shown.

In glucose minimal medium, the Δ*mdh*Δ*ldhA* mutant, which can oxidize NADH via Ndh, showed an 8% reduced growth rate, a slowed transition to the stationary phase, 21% reduced biomass formation, and a 14% reduced GUR (Figure 3A, Table 3). The slighly reduced growth rate might be due to the inability of the Δ*mdh*Δ*ldhA* mutant to form l-lactate, which is transiently formed by the wild type as NADH oxidation product and subsequently consumed again (Koch-Koerfges et al., 2012). The NAD^+^/NADH ratio (Table 4) of the Δ*mdh*Δ*ldhA* mutant (2.5 ± 0.2) was lower than in the wild type (4.7 ± 0.3), but higher than in the Δ*ndh* mutant (2.0 ± 2.0), suggesting that the enzyme couples LdhA-LldD and/or Mdh-Mqo contribute to NADH oxidation. In minimal medium with l-lactate or acetate as carbon sources, growth of the Δ*mdh*Δ*ldhA* mutant was comparable to that of the wild type (Figure 3), suggesting that both LdhA and Mdh are irrelevant for NADH oxidation on these substrates.

### Characteristics of a Δ*ndh*Δ*ldhA* Double Mutant

The Δ*ndh*Δ*ldhA* mutant, which presumably can oxidize NADH only via the Mdh-Mqo couple, showed a stronger growth defect in glucose minimal medium than the Δ*ndh* mutant (Figure 3A). The growth rate was 0.25 ± 0.1 (Δ*ndh* mutant 0.32 ± 0.1) and the final OD_600_ was 37.4 ± 2.6 (Δ*ndh* mutant 50.9 ± 1.5). In agreement with the reduced growth rate, the GUR was reduced by 17% compared to the Δ*ndh* mutant (53 ± 2 vs. 64 ± 2 nmol min^−1^ ${\text{mg}}_{\text{CDW}}^{-1}$). These results suggest that the LdhA-LldD couple plays a role in NADH oxidation on glucose besides Ndh. As expected, due to the absence of LdhA, the Δ*ndh*Δ*ldhA* mutant did not form l-lactate. Instead, the formation of acetate, succinate, and pyruvate strongly increased, with acetate being the major by-product (Table 3). This suggests that due to limited NADH reoxidation the pyruvate dehydrogenase activity and the TCA cycle activity is limited, causing an increased overflow metabolism. The NAD^+^/NADH ratio of the Δ*ndh*Δ*ldhA* mutant was 1.8 ± 0.1 and thus lower than the one of the Δ*ndh* mutant (2.0 ± 0.1).

The growth of the Δ*ndh*Δ*ldhA* mutant in acetate minimal medium was comparable to that of the wild type (Figure 3C) and the Δ*ndh* mutant (Figure 2B) and therefore LdhA is not relevant for this substrate. Interestingly, growth of the Δ*ndh*Δ*ldhA* mutant in l-lactate minimal medium was comparable to that of the wild type (Figure 3E). Thus, the additional deletion of *ldhA* abolished the positive growth effect of the *ndh* deletion on l-lactate utilization (see above). The positive influence of the *ndh* deletion on l-lactate correlated with an increased LldD activity, which was not observed in the Δ*ndh*Δ*ldhA* mutant (Table 4). This indicates that the formation of l-lactate from pyruvate by LdhA is necessary to trigger increased *lldD* expression, most likely via relieve of *lldD* repression by the transcriptional regulator LldR (Gao et al., 2008; Georgi et al., 2008).

### Characteristics of a Δ*ndh*Δ*mdh* Double Mutant

In glucose minimal medium, the Δ*ndh*Δ*mdh* mutant, which presumably can oxidize NADH only via the LdhA-LldD couple, showed a growth rate of 0.24 h^−1^, a GUR of 59 ± 3 nmol min^−1^ ${\text{mg}}_{\text{CDW}}^{-1}$, and final OD_600_ of 52.9 ± 0.4, corresponding to reductions of 38, 40, and 33% compared to the wild type (Table 3). The Δ*ndh*Δ*mdh* mutant secreted 26% more l-lactate than the wild type (Table 3), which supports the importance of LdhA for NADH oxidation. With a value of 1.3 ± 0.1, the NAD^+^/NADH ratio of the Δ*ndh*Δ*mdh* mutant was the lowest one observed in our study (Table 4). This suggests that NADH oxidation by the LdhA-LldD couple is less efficient than by Ndh or Mdh-Mqo, although the activities of LdhA and LldD were highest in the Δ*ndh*Δ*mdh* mutant with values of 0.40 and 0.31 U mg^−1^, respectively (Table 4).

In contrast to the Δ*mdh*Δ*ldhA* and Δ*ndh*Δ*ldhA* double mutants, the Δ*ndh*Δ*mdh* mutant was unable to grow with acetate or l-lactate (Figure 3). The growth defect on acetate and L-lactate could be complemented by plasmid-based expression of either *ndh* (pAN6-*ndh*) or *mdh* (pAN6-*mdh*) (data not shown). Whereas, acetate was not consumed at all by the Δ*ndh*Δ*mdh* mutant (Figure 3D), a decrease of the l-lactate concentration was observed within the first 20 h of incubation (Figure 3F) and pyruvate was secreted (Table 3). This suggests that the cells possessed LldD activity, allowing oxidation of l-lactate to pyruvate with concomitant reduction of menaquinone to menaquinol, which was then reoxidized by the terminal oxidases. The observation that pyruvate was excreted rather than further oxidized is probably caused by an insufficient pyruvate dehydrogenase activity due to the limited availability of the cofactor NAD^+^. The phenotype of the Δ*ndh*Δ*mdh* mutant suggests that NADH oxidation during growth on acetate and l-lactate requires either Ndh or the Mdh-Mqo couple, which cannot be replaced by the LdhA-LldD couple.

### Restoration of Growth on L-lactate of the Δ*ndh*Δ*mdh* Mutant

**Figure 4:**
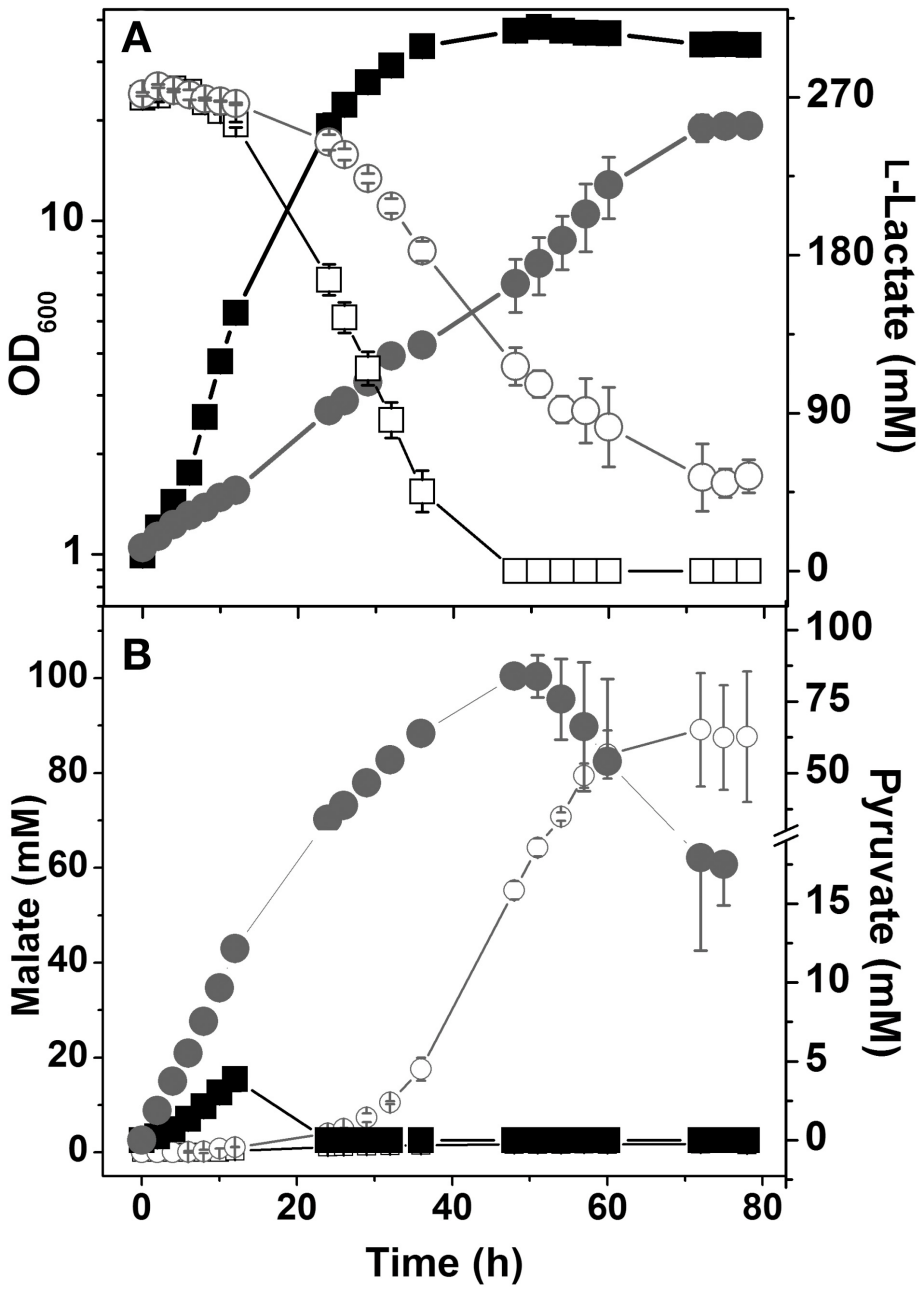
Growth and carbon source consumption **(A)** and production of organic acids **(B)** by *C. glutamicum* wild type and the mutant Δ*ndh*Δ*mdh*Δ*sugR*. **(A)** Growth (filled symbols) and l-lactate consumption (open symbols) of the wild type (■, □) and the Δ*ndh*Δ*mdh*Δ*sugR* mutant (●, ○). **(B)** Formation of pyruvate (closed symbols) and malate (open symbols) by the wild type (■, □) and Δ*ndh*Δ*mdh*Δ*sugR* mutant (●, ○). Average values and standard deviations from at least three independent cultivations are shown.

It was shown that expression of *ldhA* in *C. glutamicum* is tightly repressed by the transcriptional regulator SugR in the absence of sugars (Engels et al., 2008; Toyoda et al., 2009). As LdhA is probably the only catabolic NADH oxidation enzyme left in the Δ*ndh*Δ*mdh* mutant, repression of *ldhA* by SugR might be responsible for the inhibited growth on l-lactate. To test this hypothesis we constructed a Δ*ndh*Δ*mdh*Δ*sugR* mutant, which indeed was able to grow on l-lactate as sole carbon and energy source, although at a much lower growth rate (Figure 4A). The triple mutant secreted high amounts of pyruvate and later also malate (Figure 4B). Growth of the Δ*ndh*Δ*mdh* mutant in l-lactate minimal medium was also possible by plasmid-based expression of *ldhA* using plasmid pAN6-*ldhA* (data not shown). Thus, insufficient LdhA activity due to *ldhA* repression by SugR is an important reason for the growth defect of the Δ*ndh*Δ*mdh* mutant in L-lactate medium. Neither the Δ*ndh*Δ*mdh*Δ*sugR* mutant nor the Δ*ndh*Δ*mdh* mutant carrying pAN6-*ldhA* were able to grow on acetate (data not shown), indicating that there must be additional reasons for the inability to utilize acetate.

## Discussion

In the present work, we investigated a set of six mutants, namely Δ*ndh*, Δ*ldhA*, Δ*mdh*, Δ*mdh*Δ*ldhA*, Δ*ndh*Δ*ldhA*, and Δ*ndh*Δ*mdh*, that lack one or two of the three known catabolic NADH oxidation enzymes in *C. glutamicum* to explore their role for aerobic growth and metabolism on glucose, acetate, and l-lactate. As our attempts to construct the triple mutant Δ*ndh*Δ*mdh*Δ*ldhA* mutant failed, it appears likely that there is no further enzyme enabling catabolic NADH oxidation in *C. glutamicum* besides Ndh, Mdh, and LdhA.

In two previous studies, it was reported that an *ndh* integration mutant grows at a slightly lower rate than the wild type ATCC 13032 on glucose and acetate agar plates (Molenaar et al., 2000) and that the growth rate of an *ndh* disruption mutant of the lysozyme-sensitive strain KY9714 in glucose minimal medium was not much altered compared to the parent strain (Nantapong et al., 2004). Our studies with the in-frame deletion mutant Δ*ndh* of strain ATCC 13032 revealed a slightly reduced growth rate and biomass formation in glucose minimal medium along with reduced glucose and oxygen consumption rates. A strong effect was observed for the NAD^+^/NADH ratio, which decreased from 4.7 in the wild type to 2.0 in the Δ*ndh* mutant. As growth and the NAD^+^/NADH ratio of the strains Δ*ldhA* and Δ*mdh* in glucose minimal medium were comparable to the wild type (Table 4), our results show that Ndh is the most important enzyme for NADH oxidation during aerobic growth of *C. glutamicum* on glucose.

The observation that the *C. glutamicum* Δ*ndh* mutant did not show a stronger growth defect on glucose was explained by the existence of alternative NADH oxidation systems formed by the couples Mdh-Mqo and LdhA-LldD, which catalyze the same net reaction as Ndh, namely the oxidation of NADH and reduction of menaquinone when acting in concert (Molenaar et al., 2000; Nantapong et al., 2004). In line with this explanation, the Δ*ndh*Δ*mdh* and Δ*ndh*Δ*ldhA* double mutants showed stronger growth defects and a stronger reduction of the NAD^+^/NADH ratio than the Δ*ndh* mutant in glucose minimal medium. Therefore, neither the Mdh-Mqo couple alone nor the LdhA-LldD couple alone is able to compensate the lack of the other two NADH oxidation systems adequately. The Δ*mdh*Δ*ldhA* mutant, which still can oxidize NADH via Ndh, grew better than the Δ*ndh*Δ*mdh* and Δ*ndh*Δ*ldhA* mutants, but not as good as the wild type (Table 3), suggesting that the Mdh-Mqo and LdhA-LldD couples contribute to NADH oxidation also when Ndh is present.

Growth on l-lactate was improved by the *ndh* deletion (Figures 2C,D). This positive effect is presumably due to a 2-fold increased activity of LldD (Table 4), which was observed also previously in the study of the *ndh* mutant of strain KY9714 (Nantapong et al., 2005). It has been shown that expression of the cg3226-*lldD* operon is regulated by the FadR-type transcriptional regulator LldR, which represses the two genes in the absence of l-lactate (Gao et al., 2008; Georgi et al., 2008). The gene cg3226 encodes a putative l-lactate permease, which is not essential for growth on l-lactate, however (Stansen et al., 2005). The reason for the increased LldD activity in the Δ*ndh* mutant is most likely the increased formation of l-lactate (Table 3), which relieves repression of the cg3226-*lldD* operon by LldR. In line with this explanation, the additional deletion of *ldhA* in the Δ*ndh* strain reversed the improved growth and the increased LldD activity (Figures 3C,D, Tables 2, 3). The reason why even l-lactate-adapted wild-type cells grew not as good as the Δ*ndh* mutant (Figure 2C) is not clear yet. The absence of Ndh might allow for an increased availability of menaquinone as electron acceptor of LldD.

Compared to glucose, growth on acetate of the strains Δ*ndh*, Δ*ndh*Δ*ldhA*, and Δ*mdh*Δ*ldhA* was only minimally affected (Figures 2, 3, Table 3). This difference can be explained by the fact that glucose oxidation is coupled to the formation of up to 6 NADH/glucose, while acetate oxidation is linked to the formation of only 1 NADH/acetate (Figure 1). Thus, glucose oxidation has a much higher NADH reoxidation demand than lactate or acetate oxidation. As essentially no l-lactate is formed during growth on acetate (Table 3) and *ldhA* is repressed by SugR, the LdhA-LldD cycle is probably not active and NADH oxidation is accomplished via the Mdh-Mqo cycle in the mutants Δ*ndh* and Δ*ndh*Δ*ldhA* and via Ndh in strain Δ*mdh*Δ*ldhA*. In agreement with this interpretation, the Δ*ndh*Δ*mdh* strain was unable to grow on acetate (Figure 3).

In summary, our results show that *C. glutamicum* uses a set of three systems for NADH oxidation coupled to menaquinone reduction. Ndh is the dominant enzyme, but the Mdh-Mqo couple and LdhA-LldD couples complement or substitute it and thereby enable NADH oxidation in situations with high catabolic fluxes or a defective Ndh enzyme. Such backup systems exist not only for NADH oxidation, but also for menaquinol oxidation, where cytochrome *bd* oxidase can substitute or complement the cytochrome *bc*_1_-*aa*_3_ supercomplex. This redundancy likely contributes to the metabolic flexibility and the survival of the organism in nature.

## Data Availability Statement

The original contributions presented in the study are included in the article/supplementary material, further inquiries can be directed to the corresponding author/s.

## Author Contributions

MB and AK-K designed the study. TM and AK-K performed the experiments and prepared the figures and tables. MB wrote the manuscript. All authors contributed to the interpretation of the data.

## Conflict of Interest

The authors declare that the research was conducted in the absence of any commercial or financial relationships that could be construed as a potential conflict of interest.
